# Determinants of perceived stress among adolescents during wartime in Ukraine

**DOI:** 10.3389/fped.2026.1838159

**Published:** 2026-04-22

**Authors:** Tetyana Hariyan, Tymur Hariyan, Oksana Boyarchuk

**Affiliations:** 1 Department of Children’s Diseases and Pediatric Surgery, I.Horbachevsky Ternopil National Medical University, Ternopil, Ukraine; 2 Ternopil Academic Lyceum “Ukrainian Gymnasium” Named After Ivan Franko, Ternopil, Ukraine

**Keywords:** adolescents, mental health, perceived stress, physical activity, sleep disturbances, Ukraine, war exposure

## Abstract

**Introduction:**

Adolescents living in war-affected environments are exposed to prolonged and multifactorial stressors that may adversely impact their mental health. In Ukraine, the ongoing war has created conditions of chronic stress even among those not directly exposed to combat. Understanding the determinants of perceived stress in this population is essential for developing targeted preventive strategies. The aim of this study was to assess the level of perceived stress among adolescents and to examine its associations with demographic characteristics, sleep patterns, and physical activity

**Methods:**

A cross-sectional study was conducted among adolescents to evaluate levels of perceived stress and its associated factors. Perceived stress was assessed using the 10-item Perceived Stress Scale (PSS-10). Data on sleep duration, sleep quality, physical activity, and selected health-related factors were collected using self-reported questionnaires. Correlation and comparative analyses were performed to evaluate associations between variables.

**Results:**

The study included 198 participants. A high prevalence of perceived stress was observed, with 52.0% of participants reporting moderate and 37.4% high levels of perceived stress, while only 10.6% demonstrated low stress levels. A significantly higher proportion of females reported high stress levels compared to males (21.5% vs. 5.1%, *p* < 0.0005). Poor sleep quality and difficulties initiating sleep were also associated with higher stress levels (*p* < 0.05). Adolescents sleeping more than 8 h per night were significantly more likely to report low stress levels compared to those sleeping less than 8 h (33.3% vs. 11.8%, *p* = 0.0006). Higher levels of physical activity were associated with lower stress (*p* < 0.05). Participants engaging in physical activity ≥5 days per week more frequently reported low stress compared to less active peers (32.7% vs. 9.4%, *p* < 0.001). No significant association was found between perceived stress and the presence of chronic diseases.

**Conclusion:**

Adolescents in a war-affected setting demonstrate a markedly elevated level of perceived stress. Sleep and physical activity are significantly but modestly associated with perceived stress. These findings support the integration of lifestyle-focused and psychosocial interventions into adolescent health strategies to help mitigate the long-term impact of chronic stress.

## Introduction

War represents a major public health challenge with profound and long-term consequences for children and adolescents. In Ukraine, the relevance of this issue has increased dramatically since the onset of the full-scale invasion, which has become a powerful and prolonged psychological stressor for the pediatric population (1). Persistent threats to safety, disruption of educational processes (including remote and hybrid learning), reduced social interactions, emotional strain within families, and continuous exposure to distressing information collectively contribute to elevated stress levels among children and adolescents (1, 2). Adolescents constitute a particularly vulnerable group due to the ongoing development of emotional regulation, cognitive control, and coping mechanisms (2).

War exposure encompasses both acute traumatic events, such as missile and drone attacks, forced displacement, and loss of loved ones and chronic stressors related to sustained insecurity, uncertainty about the future, disruption of daily routines, and breakdown of social connections (3–5). The cumulative and prolonged nature of these stressors is associated with an increased risk of persistent psychological distress and maladaptive outcomes (3, 4). Beyond its direct psychological effects, war also indirectly disrupts key lifestyle factors in children and adolescents, including daily routines, physical activity, sleep patterns, nutrition, and access to healthcare and supportive resources, which may further contribute to the development and maintenance of chronic stress (4, 6).

Importantly, the impact of war extends beyond immediate trauma and includes continuous exposure to chronic stressors such as air raid alarms, repeated attacks, and prolonged uncertainty. Adolescents living in conflict settings report higher levels of psychological distress, including depressive symptoms, anxiety, and increased risk of suicidal ideation and behaviors (7, 8, 10). In addition, prolonged exposure to war conditions may affect identity development, social functioning, and behavioral regulation, leading to social withdrawal and engagement in maladaptive coping strategies (9, 10). While a substantial body of research has focused on post-traumatic stress disorder and acute trauma, considerably less attention has been paid to chronic perceived stress in adolescents living under prolonged war conditions, highlighting a critical gap in the current literature.

Assessment of stress in adolescents should be complemented by the evaluation of modifiable behavioral and lifestyle factors. In war-affected populations, stress often manifests through a combination of psychological, emotional, and somatic symptoms, including sleep disturbances, impaired concentration, emotional instability, and psychosomatic complaints (1, 6, 11). These manifestations may be further exacerbated by disruptions in daily structure and reduced access to supportive environments.

Particularly vulnerable are children with chronic health conditions, for whom war-related disruptions in healthcare access, treatment continuity, and social support may exacerbate both physical and psychological burden (12, 13). In such populations, the combined effects of chronic illness and prolonged external stressors may lead to increased vulnerability to stress and poorer health outcomes, underscoring the need for targeted attention in research and clinical practice.

Since 2022, Ukrainian adolescents have been living under conditions of prolonged war. Despite the magnitude and chronic nature of the ongoing crisis, systematic evidence on stress levels and their associated factors among adolescents remains limited. Adolescent stress is a multifactorial phenomenon shaped by biological, psychological, and social determinants, with significant implications for both immediate well-being and long-term health trajectories (14).

In this context, the level of perceived stress may be influenced by demographic characteristics, health status, and modifiable lifestyle factors, including sleep quality and duration, physical activity, and behavioral patterns. Identifying these associations is essential for understanding potential intervention targets.

Given the well-established negative effects of chronic stress, systematic assessment of stress levels among adolescents in war settings, along with identification of associated factors, is essential for early detection of at-risk groups and the development of targeted preventive and intervention strategies aimed at reducing stress burden and enhancing adaptive capacity.

Therefore, the aim of this study was to assess the level of perceived stress among school-aged adolescents during wartime and to determine the influence of selected behavioral factors on its development.

## Materials and methods

### Study design and setting

This cross-sectional study was conducted using an anonymous online survey among high school students in Ternopil, Ukraine. Data collection took place between October 1 and November 30, 2025, during the ongoing full-scale war, which at the time had lasted 3 years and 8 months.

Although the Ternopil region is not a frontline area, it remains regularly exposed to missile and drone attacks. Many participants reported indirect exposure to war-related stressors, including having family members involved in military service and frequent exposure to war-related information and community losses. This context was considered an important background factor influencing stress perception.

### Participants

The study included students in grades 10–11 from Ternopil Academic Lyceum “Ukrainian Gymnasium” named after Ivan Franko.

Inclusion criteria were age consistent with grades 10–11 (typically 15–17 years), voluntary participation, provision of informed consent. Participation was anonymous and voluntary, with no incentives provided.

### Ethical considerations

The study was conducted in accordance with ethical principles for research involving human participants. Participation was voluntary, and informed consent was obtained from all participants as well as from their parents or legal guardians prior to enrollment. An information sheet describing the purpose of the study, confidentiality, and data use was provided at the beginning of the survey. No personally identifiable information was collected, ensuring full anonymity and confidentiality of responses.

### Data collection

Data were collected using a structured, self-administered questionnaire developed in Google Forms. The questionnaire consisted of the following sections:

1. General information and consent
2. Sociodemographic characteristics (age, sex, place of residence, family structure)
3. Perceived stress assessment (PSS-10)
4. Behavioral and lifestyle factors, including: sleep duration, sleep quality, difficulty falling asleep, physical activity, consumption of caffeine and alcohol.
5. Health-related information (presence of chronic diseases).

### Assessment of perceived stress (PSS-10)

Perceived stress was assessed using the 10-item Perceived Stress Scale (PSS-10), originally developed by Cohen et al. (15). The scale measures the extent to which individuals perceive their lives as unpredictable, uncontrollable, and overwhelming during the past month.

Each item is scored on a 5-point Likert scale ranging from 0 (“never”) to 4 (“very often”), resulting in a total score ranging from 0 to 40. Items 4, 5, 7, and 8 are positively stated and were reverse-coded prior to analysis. Higher total scores indicate higher levels of perceived stress.

The Ukrainian-language version of the Perceived Stress Scale (PSS-10) was used in this study. The scale has been previously translated, culturally adapted, and psychometrically validated in Ukrainian populations, demonstrating good internal consistency and construct validity (16). Therefore, it can be considered a reliable tool for assessing perceived stress in Ukrainian-speaking respondents.

For descriptive interpretation, stress levels were categorized as follows (based on commonly used thresholds in the literature): low stress: 0–13; moderate stress: 14–26; high stress: 27–40.

### Definition of behavioral variables

To assess associations between lifestyle factors and perceived stress, key behavioral variables were categorized based on established recommendations:

Sleep duration was categorized using a threshold of 8 h per night, in accordance with international recommendations for adolescents, which suggest 8–10 h of sleep for optimal health and functioning. The use of a binary cut-off for sleep duration was applied to simplify statistical analysis and to identify adolescents at risk of insufficient sleep. Sleep quality was assessed using a single self-reported item with five ordinal response categories: “slept very badly,” “slept badly,” “slept normally,” “slept well,” and “slept very well (fully slept).” Responses were coded on a scale from 1 to 5, with higher scores indicating better perceived sleep quality. This variable was analyzed as an ordinal measure in the statistical analysis.

Physical activity was categorized based on frequency per week: ≥5 days per week and <5 days per week. This threshold was selected in line with WHO recommendations, which suggest that adolescents should engage in moderate-to-vigorous physical activity on most days of the week (ideally daily), making ≥5 days a reasonable proxy for adequate activity.

### Statistical analysis

Statistical analysis was performed using STATISTICA version 10 (StatSoft Inc., USA).

Descriptive statistics were used to summarize the data. Continuous variables were presented as mean ± standard deviation (SD) and categorical variables were presented as frequencies and percentages. Comparisons between groups were performed using the chi-square (*χ*²) test. A *p*-value <0.05 was considered statistically significant.

Correlations were assessed using Spearman's rank correlation coefficient due to the ordinal nature of several variables. For each correlation, both the correlation coefficient (r) and the corresponding *p*-value were reported. Associations between PSS-10 scores and demographic or clinical variables were analyzed to identify potential factors influencing stress levels.

## Results

### Study population characteristics

A total of 204 students participated in the survey. However, 8 respondents were excluded from the analysis: 4 did not provide informed consent and 4 submitted incomplete or inconsistent responses. Therefore, the final analysis included 198 participants.

The demographic characteristics of the study population are presented in Table 1.

**Table 1:** Socio-demographic characteristics of the studied adolescents (*n* = 198).

Answers for questions about:		*n*	%
Age	15	62	31.3
	16	135	68.2
	17	1	0.5
Gender	Female	93	47.0
	Male	98	49.5
	Don't want to answer	7	3.5
Grade	11	100	50.5
	10	98	49.5
Place of residence	Urban	164	82.8
	Rural	34	17.2
Living with both parents	Yes	156	78.8
	No	42	21.2

The majority of respondents were 16 years old (68.2%), with no significant differences between students in grades 10 and 11 or by gender.

Most respondents resided in urban areas (82.8%) and lived with both parents (78.8%,).

### Responses to the PSS-10 items

The distribution of responses to individual PSS-10 items is presented in Supplementary Table S1 (Supplementary material). For 9 out of 10 items, the most frequently selected response was “sometimes,” with proportions ranging from 30.3% to 43.9%.

An exception was the item assessing confidence in one's ability to cope with personal problems, where 44.4% of respondents selected “fairly often,” suggesting a relatively preserved sense of coping capacity despite exposure to stress. However, 30.3% reported this feeling only “sometimes,” indicating variability in perceived control.

### Lifestyle and health-related characteristics

Lifestyle and health-related variables are summarized in Table 2. The mean self-reported sleep duration was 6.8 ± 1.3 h per night. Most respondents rated their sleep quality as “average” (38.9%), while a substantial proportion reported difficulties falling asleep (41.4%). The majority of adolescents (77.3%) reported sleeping less than 8 h per night, which was significantly higher compared to those who slept ≥8 h (*p* < 0.0001).

**Table 2:** Lifestyle and health-related characteristics of the studied adolescents (*n* = 198).

Question	Answer options	*n*	%
**Block of questions about sleep**			
1. How many hours do you sleep on weekdays?	Number from 3 to 14	Mean 6.8	–
2.Sleep quality	Slept very badly	12	6,1
	Slept badly	36	18,2
	Slept normally	77	38,9
	Slept well	57	28,8
	Slept very well (fully slept)	16	8,1
3. Do you have trouble falling asleep?	Yes	82	41,4
	No	116	58,6
How many days a week do you engage in physical activity for more than 30 min?	More than 5	61	30,8
	Less than 5	137	69,2
Do you drink coffee or energy drinks?	No	145	73,2
	Yes	53	26,8
Do you smoke or drink alcohol?	No	150	75,8
	Sometimes	48	24,3
	Regular	0	0
Do you have any chronic diseases?	No	180	90,9
	Yes	18	9,1

Regarding physical activity, the majority of participants (69.2%) reported engaging in ≥30 min of physical activity on fewer than 5 days per week, which was significantly higher than the proportion of adolescents meeting the recommended level of physical activity (*p* < 0.0001).

Most respondents reported no daily consumption of caffeine or energy drinks (73.2%) and no use of tobacco or alcohol (75.8%). Chronic diseases were reported by 9.1% of participants.

### Perceived stress levels according PSS-10

**Figure 1:**
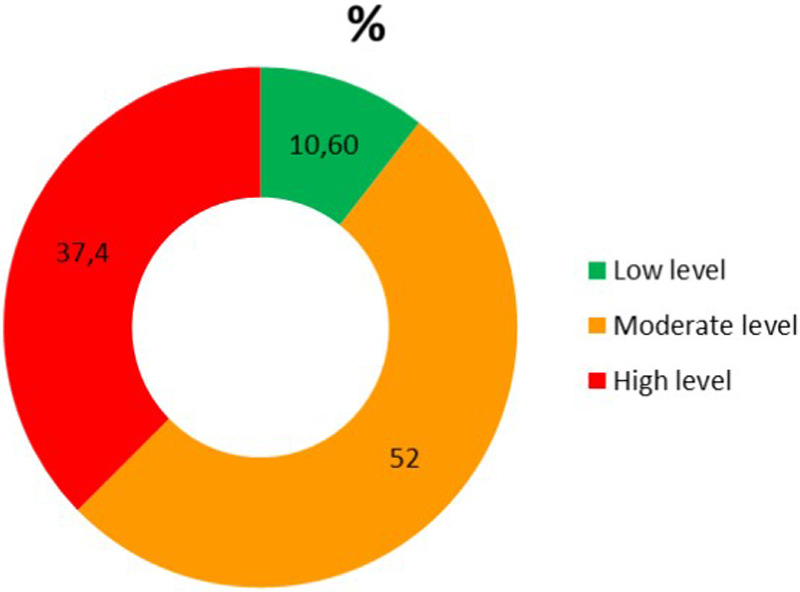
Percentage distribution of perceived stress levels among adolescents based on the PSS-10 scale (*n* = 198).

The distribution of perceived stress levels is presented in Figure 1.

The mean PSS-10 score was 19.7 ± 5.9 (range 7–40). Most students demonstrated a moderate level of perceived stress (52.0%). A high level of stress was observed in 37.4% of respondents, while only 10.6% had low stress levels.

Overall, the prevalence of elevated stress (moderate and high levels) was substantial, suggesting a considerable stress burden in this population.

### Factors associated with perceived stress

Associations between perceived stress levels and selected sociodemographic and behavioral factors are presented in Table 3. Age showed a trend toward significance for low stress levels, with younger adolescents (15 years) more likely to report low stress compared to 16-year-olds (24.2% vs. 13.3%, *p* = 0.0580), although this did not reach statistical significance. No significant differences in stress levels were observed between students in grades 10 and 11. Similarly, no statistically significant associations were found between stress levels and place of residence (urban vs. rural) or living with both parents.

**Table 3:** Distribution of perceived stress levels (PSS-10) among adolescents according to sociodemographic and behavioral characteristics (*n* = 198).

Characteristic	Low level	Moderate level	High level	Total
	*n* (%)	*n* (%)	*n* (%)	*n* (%)
**Grade**				
10	18 (18.4)	66 (67.3)	14 (14.3)	98 (49.5)
11	15 (15.0)	73 (73.0)	12 (12.0)	100 (50.5)
Р	0.5250	0.3845	0.6340	0.8407
**Age**				
15	15 (24.2)	41 (66.1)	6 (9.7)	62 (31.3)
16	18 (13.3)	97 (71.9)	20 (14.8)	135 (68.2)
Р	0.0580	0.4154	0.3225	**<0.0001**
**Gender**				
Female	11 (11.8)	62 (66.7)	20 (21.5)	93 (47.0)
Male	22 (22.4)	71 (72.4)	5 (5.1)	98 (49.5)
Р	0.0523	0.3851	**<0.0005**	0.6089
**Place of residence**				
Urban	27 (16.5)	114 (69.5)	23 (14.0)	164 (82.8)
Rural	6 (17.7)	25 (73.5)	3 (8.8)	34 (17.2)
Р	0.8662	0.6411	0.4138	**<0.0001**
**Living with both parents**				
Yes	26 (16.7)	113 (72.4)	17 (10.9)	156 (78.8)
No	7 (16.7)	26 (61.9)	9 (21.4)	42 (21.2)
Р	1.000	0.1853	0.0729	**<0.0001**
**Sleep duration**				
<8 h	18 (11.8)	112 (73.2)	23 (15.0)	153 (77.3)
≥8 h	15 (33.3)	27 (60.0)	3 (6.7)	45 (22.7)
P	**0.0006**	0.0887	0.1441	**<0.0001**
**Difficulty falling asleep**				
Yes	6 (7.3)	62 (75.6)	14 (17.1)	82 (41.4)
No	27 (23.3)	77 (66.4)	12 (10.3)	116 (58.6)
P	**0.0065**	0.1619	0.1673	**0.0006**
**Physical activity per week**				
More than 5 days	20 (32.7)	34 (55.7)	7 (11.5)	61 (30.8)
Less than 5 days	13 (9.4)	105 (76.6)	19 (14.0)	137 (69.2)
Р	**<0.001**	**0.0030**	0.6453	**<0.0005**
**Chronic diseases**				
Present	4 (22.2)	10 (55.6)	4 (22.2)	18 (9.1)
Absent	29 (16.1)	129 (71.7)	22 (12.2)	180 (90.9)
Р	0.5071	0.1542	0.2310	**<0.0001**
**Drinking coffee or energy drinks**				
No	29 (20.0)	97 (66.9)	19 (13.1)	145
Yes	4 (7.6)	42 (79.2)	7 (13.2)	53
P	**0.0374**	0.0926	0.9847	**<0.0001**
**Smoking or drinking alcohol**				
No	26 (17.3)	103 (68.7)	21 (14.0)	150 (75.8)
Sometimes	7 (14.6)	36 (75.0)	5 (10.4)	48 (24.2)
P	1.000	0.4037	0.5223	**<0.0001**

Statistically significant values are highlighted in bold.

Gender differences approached statistical significance for low stress levels and were highly significant for high stress levels. A significantly higher proportion of females reported high stress levels compared to males (21.5% vs. 5.1%, *p* < 0.0005), indicating that high stress was approximately four times more frequent among female students.

Sleep-related factors demonstrated important associations with perceived stress. Adolescents who reported sleeping ≥8 h per night were significantly more likely to have low stress levels compared to those sleeping <8 h (33.3% vs. 11.8%, *p* = 0.0006). Low stress levels were more frequently observed among adolescents who did not report difficulty falling asleep compared to those who did (23.3% vs. 7.3%, *p* = 0.0065).

Participants engaging in physical activity more than 5 days per week were significantly more likely to report low stress levels compared to those with lower activity (32.7% vs. 9.4%, *p* < 0.001). Conversely, moderate stress levels were more common among less active adolescents (76.6% vs. 55.7%, *p* = 0.0030). These findings suggest that regular physical activity is associated with lower perceived stress.

No statistically significant differences in stress levels were observed between participants with and without chronic diseases.

Consumption of coffee or energy drinks was associated with stress levels. Adolescents who did not consume these beverages were more likely to report low stress compared to those who did (20.0% vs. 7.6%, *p* = 0.0374), while no significant differences were observed for moderate or high stress levels. Finally, no statistically significant associations were found between stress levels and smoking or alcohol consumption.

Overall, the findings indicate that female gender, insufficient sleep, difficulty falling asleep, lower physical activity, and consumption of caffeinated beverages are associated with higher perceived stress among adolescents, whereas grade, place of residence, family structure, and chronic disease status were not significantly associated with stress levels.

Correlation analysis revealed several statistically significant associations between total PSS-10 scores and selected variables. A weak positive correlation was found between stress level and female sex (*r* = 0.264, *p* < 0.05). Sleep duration was negatively correlated with stress (*r* = −0.260, *p* < 0.05), indicating that shorter sleep duration was associated with higher stress levels. Sleep quality demonstrated a moderate negative correlation with stress (*r* = −0.333, *p* < 0.05), suggesting that poorer sleep quality was associated with higher perceived stress. Difficulties with sleep initiation showed a moderate positive correlation with stress (*r* = 0.324, *p* < 0.05), indicating a close relationship between sleep disturbances and psychological stress. Physical activity was weakly negatively correlated with stress (*r* = −0.170, *p* < 0.05), with lower activity levels associated with higher stress. Other variables did not show statistically significant correlations with PSS-10 scores.

## Discussion

The present study reveals a high prevalence of perceived stress among adolescents in the context of the ongoing war in Ukraine, with more than 89% of participants reporting moderate or high stress levels and over one-third experiencing high perceived stress. These findings indicate a substantial psychological burden in this population and suggest that prolonged exposure to war-related stressors may significantly compromise adolescents' adaptive capacity and emotional well-being.

Importantly, even in the absence of direct exposure to combat, adolescents are continuously affected by indirect but persistent stressors, including air raid alarms, disruption of daily routines, remote learning, information overload, and social isolation. The cumulative and chronic nature of these stressors likely contributes to sustained activation of stress-response systems, increasing vulnerability to emotional dysregulation and mental health problems. These observations are consistent with previous research demonstrating elevated levels of psychological distress among youth in war-affected settings (1, 10, 17).

Importantly, this study adds to the existing literature by providing data from a population exposed to prolonged war-related stress in a real-world setting, where indirect but persistent stressors predominate. Unlike many previous studies conducted in stable environments, our findings highlight the relevance of modifiable lifestyle factors even under conditions of chronic external stress.

The prevalence of high perceived stress observed in this study exceeds that reported in adolescents from non-war environments, such as the findings by Anjum et al. (18), where high stress levels were considerably less frequent. This discrepancy may be explained by differences in the intensity, chronicity, and unpredictability of stress exposure, emphasizing the unique and pervasive impact of war-related conditions on adolescent mental health.

In line with contemporary stress models, perceived stress reflects not only external circumstances but also individual appraisal processes and coping resources (2, 3). This highlights the importance of considering psychological resilience, family support, and social context when interpreting stress responses in adolescents. Variability in these factors may partially explain differences in stress levels across populations and individuals exposed to similar stressors.

A statistically significant gender difference was identified, with girls demonstrating higher levels of perceived stress compared to boys. This finding is consistent with a substantial body of literature indicating greater vulnerability to stress among female adolescents (18). Potential explanations include differences in emotional processing, coping strategies, hormonal influences, and social expectations. However, the evidence remains heterogeneous, with some studies reporting no significant gender differences or even higher stress levels among males (19, 20), suggesting that gender effects may be context-specific and influenced by sociocultural and environmental factors.

One of the most important findings of this study is the association between lifestyle factors, particularly sleep and physical activity and perceived stress. Both shorter sleep duration and poorer sleep quality were statistically significantly but modest correlated with higher stress levels. These findings are in agreement with previous studies demonstrating that insufficient and disrupted sleep is is associated with increased emotional distress and impaired stress regulation in adolescents, likely through alterations in neuroendocrine stress-response systems and emotional processing (21–23).

The relationship between sleep and stress appears to be bidirectional. Elevated stress levels may interfere with sleep initiation and maintenance, leading to fragmented or insufficient sleep, while sleep deprivation exacerbates emotional reactivity and reduces the capacity to cope with stress. This reciprocal interaction may result in a self-perpetuating cycle, particularly in high-stress environments such as those associated with armed conflict.

Notably, adolescents who reported sleeping at least 8 h per night were more likely to have lower stress levels, supporting existing recommendations regarding optimal sleep duration for this age group. Adequate sleep is essential for neurocognitive functioning, emotional regulation, and resilience to stress and its disruption may play a critical role in the development and maintenance of stress-related conditions (24).

Physical activity also emerged as a significant protective factor. Higher levels of physical activity were associated with lower perceived stress, which is consistent with evidence indicating that regular exercise reduces stress, anxiety, and depressive symptoms while improving overall psychological well-being (25, 26). The beneficial effects of physical activity are likely mediated through multiple pathways, including neurobiological mechanisms (endorphin release), improved sleep quality, and enhanced self-regulation and coping capacity (21, 27, 28).

In contrast, no statistically significant association was found between perceived stress and the presence of chronic diseases. This finding should be interpreted with caution, as it may be influenced by the relatively small number of participants with chronic conditions, heterogeneity of diagnoses, and potential adaptation mechanisms. Adolescents with chronic illnesses may develop effective coping strategies and receive increased social or medical support, which could mitigate the impact of their condition on perceived stress.

Overall, the findings of this study underscore the potential role of modifiable lifestyle factors in shaping adolescents’ stress levels, even in the context of severe and prolonged external stressors such as war. While macro-level factors cannot be easily altered, interventions targeting sleep hygiene and physical activity represent feasible and potentially effective strategies for improving mental health outcomes in this population.

At the same time, the relatively modest strength of the observed associations should be considered when interpreting these findings. This may be partly explained by potential psychological adaptation to prolonged stress exposure. Adolescents living in a war-affected environment may develop coping mechanisms that attenuate the impact of individual lifestyle factors on perceived stress. However, this assumption requires further investigation, as adaptation processes may vary across individuals and contexts.

From a clinical and public health perspective, the integration of simple screening tools, such as PSS-10, with basic assessments of lifestyle behaviors may facilitate early identification of adolescents at increased risk of stress-related problems. Such approaches could support the development of targeted, non-pharmacological interventions aimed at strengthening resilience and promoting psychological well-being.

The findings demonstrate that adolescents living in a war-affected environment experience a substantially elevated level of perceived stress, reflecting the profound and ongoing impact of chronic external stressors on mental health. Gender differences suggest increased vulnerability among girls, while lifestyle factors, particularly sleep and physical activity are statistically significantly associated with perceived stress.

Adequate sleep duration and quality, along with regular physical activity, are consistently associated with lower stress levels, highlighting their protective role even under conditions of prolonged adversity. These results emphasize the importance of integrating behavioral and psychosocial approaches in strategies aimed at improving adolescent well-being.

Taken together, the study supports the need for early identification of stress in adolescents and the implementation of accessible, non-pharmacological interventions focused on sleep hygiene, physical activity, and resilience-building. Addressing these factors may play a role in reducing the long-term mental health consequences of chronic stress exposure in this vulnerable population.

### Strengths and limitations

This study has several strengths that should be acknowledged. First, it addresses a highly relevant and underexplored topic by examining perceived stress among adolescents living in an active war context, where evidence remains limited. Second, the study evaluates not only stress levels but also modifiable behavioral factors, such as sleep and physical activity, providing clinically and public health–relevant insights into potential targets for intervention. Third, the use of a widely recognized and validated instrument, the Perceived Stress Scale (PSS-10), allows for comparability with international studies. Additionally, the inclusion of multiple lifestyle and demographic variables enables a more comprehensive assessment of factors associated with stress in this population.

However, several limitations should be considered when interpreting the findings. First, the cross-sectional design does not allow for causal inferences regarding the relationships between perceived stress and associated factors such as sleep and physical activity. The observed associations may be bidirectional, particularly in the case of stress and sleep, and longitudinal studies are needed to clarify the directionality of these relationships. Second, the data were based on self-reported measures, which may be subject to recall bias and social desirability bias. Participants may have underreported or overreported certain behaviors, including sleep duration, physical activity, or perceived stress levels. Third, the assessment of stress was conducted using the Perceived Stress Scale (PSS-10), which reflects subjective appraisal rather than objective physiological stress. While this is consistent with contemporary stress models, it may limit direct comparisons with studies using biological markers of stress. Additionally, sleep quality was assessed using a single self-reported item, which does not capture its multidimensional nature and may limit the depth of interpretation.

This study was conducted during a period of ongoing war; however, individual exposure to war-related factors (such as displacement, personal loss, or family involvement in military activities) was not directly assessed. Therefore, war-related influences were not included as analytical variables, and the findings should be interpreted within this contextual background rather than as direct consequences of war exposure.

## Conclusion

This study provides evidence of a markedly elevated burden of perceived stress among adolescents living in a war-affected environment and suggests that behavioral factors, including sleep and physical activity, are significantly but modestly associated with stress levels. These findings support the need for comprehensive, context-sensitive strategies that combine psychosocial support with lifestyle-oriented interventions to mitigate the long-term impact of chronic stress on adolescent health. Promoting healthy sleep patterns and regular physical activity may be considered important components of preventive and supportive programs for youth exposed to prolonged stress conditions.

## Data Availability

The raw data supporting the conclusions of this article will be made available by the authors, without undue reservation.
