# Supplementary material for: Determinants of perceived stress among adolescents during wartime in Ukraine
Source: Front Pediatr. 2026 Apr 22;14:1838159. doi: 10.3389/fped.2026.1838159 (PMC13144074; doi:10.3389/fped.2026.1838159)
Supplement: Supplementary file 1 [file Table1.docx]

Table S1. **Responses to the Perceived Stress Scale (PSS-10) items**

Participants indicated how often they experienced each situation during the past month.

| Question | Варіанти відповідей | n | % |
| --- | --- | --- | --- |
| 1. How often have you been upset because of something that happened unexpectedly? | 0 = Never | 7 | 3,5 |
|  | 1 = Almost never | 41 | 20,7 |
|  | 2 = Sometimes | 87 | 43,9 |
|  | 3 = Fairly often | 42 | 21,2 |
|  | 4 = Very often | 21 | 10,6 |
| 2. How often have you felt that you were unable to control the important things in your life? | 0 = Never | 10 | 5,1 |
|  | 1 = Almost never | 50 | 25,3 |
|  | 2 = Sometimes | 64 | 32,3 |
|  | 3 = Fairly often | 51 | 25,8 |
|  | 4 = Very often | 23 | 11,6 |
| 3. How often have you felt nervous and “stressed”? | 0 = Never | 6 | 3,0 |
|  | 1 = Almost never | 34 | 17,2 |
|  | 2 = Sometimes | 72 | 36,4 |
|  | 3 = Fairly often | 52 | 26,3 |
|  | 4 = Very often | 34 | 17,2 |
| 4. How often have you felt confident about your ability to handle your personal problems? | 0 = Never | 4 | 2,0 |
|  | 1 = Almost never | 19 | 9,6 |
|  | 2 = Sometimes | 60 | 30,3 |
|  | 3 = Fairly often | 88 | 44,4 |
|  | 4 = Very often | 27 | 13,6 |
| 5. How often have you felt that things were going your way? | 0 = Never | 2 | 1,0 |
|  | 1 = Almost never | 25 | 12,6 |
|  | 2 = Sometimes | 84 | 42,4 |
|  | 3 = Fairly often | 63 | 31,8 |
|  | 4 = Very often | 24 | 12,1 |
| 6. How often have you found that you could not cope with all the things that you had to do? | 0 = Never | 8 | 4,0 |
|  | 1 = Almost never | 31 | 15,7 |
|  | 2 = Sometimes | 74 | 37,4 |
|  | 3 = Fairly often | 55 | 27,8 |
|  | 4 = Very often | 30 | 15,2 |
| 7. How often have you been able to control irritations in your life? | 0 = Never | 6 | 3,0 |
|  | 1 = Almost never | 26 | 13,1 |
|  | 2 = Sometimes | 82 | 41,4 |
|  | 3 = Fairly often | 67 | 33,8 |
|  | 4 = Very often | 17 | 8,6 |
| 8. How often have you felt that you were on top of things? | 0 = Never | 1 | 0,5 |
|  | 1 = Almost never | 21 | 10,6 |
|  | 2 = Sometimes | 82 | 41,4 |
|  | 3 = Fairly often | 72 | 6,4 |
|  | 4 = Very often | 22 | 11,1 |
| 9. How often have you been angered because of things that were outside of your control? | 0 = Never | 10 | 5,0 |
|  | 1 = Almost never | 30 | 15,2 |
|  | 2 = Sometimes | 61 | 30,8 |
|  | 3 = Fairly often | 59 | 29,8 |
|  | 4 = Very often | 38 | 19,2 |
| 10. How often have you felt difficulties were piling up so high that you could not overcome them? | 0 = Never | 22 | 11,1 |
|  | 1 = Almost never | 45 | 27,7 |
|  | 2 = Sometimes | 65 | 32,8 |
|  | 3 = Fairly often | 41 | 20,7 |
|  | 4 = Very often | 25 | 12,6 |
